# Substrate interaction inhibits γ-secretase production of amyloid-β peptides†

**DOI:** 10.1039/c9cc09170j

**Published:** 2020-02-27

**Authors:** Jing Zhao, Yuanyuan Xiao, Xinyue Liu, Soohyun Kim, Xianzhong Wu, Marilia Barros, Ran Zhuang, Xuben Hou, Yingkai Zhang, Nikolaos K. Robakis, Yue-Ming Li, Jonathan S. Dordick, Iban Ubarretxena-Belandia, Chunyu Wang

**Affiliations:** a Center for Biotechnology and Interdisciplinary Studies, Rensselaer Polytechnic Institute, Troy, NY 12180, USA.; b Center for Molecular Biology and Genetics of Neurodegeneration, Departments of Psychiatry and Neuroscience, Icahn School of Medicine at Mount Sinai, New York, NY 10029, USA; c Chemical Biology Program, Memorial Sloan Kettering Cancer Center, New York, NY 10065, USA; d Department of Medicinal Chemistry and Key Laboratory of Chemical Biology of Natural Products (MOE), School of Pharmacy, Shandong University, Jinan, Shandong 250012, China; e Department of Chemistry, New York University, New York, NY 10003, USA; f Department of Biological Sciences, Rensselaer Polytechnic Institute, Troy, NY 12180, USA; g Department of Chemical and Biological Engineering, Rensselaer Polytechnic Institute, Troy, NY 12180, USA; h Department of Pharmacological Sciences, Icahn School of Medicine at Mount Sinai, New York, NY 10029, USA; i Instituto Biofisika (UPV/EHU, CSIC), University of the Basque Country, E-48940, Leioa, Spain; j Ikerbasque, Basque Foundation for Science, 48013, Bilbao, Spain; k Department of Chemistry and Chemical Biology, Rensselaer Polytechnic Institute, Troy, NY 12180, USA

## Abstract

Combining NMR, mass spectrometry, AlphaLISA and cell assays, we discovered a compound C1 that binds C-terminal juxtamembrane lysines at the transmembrane domain of the amyloid precursor protein (APPTM) and inhibits γ-secretase production of amyloid-β with μM IC_50_. Our work suggests that targeting APPTM is a novel and viable strategy in AD drug discovery.

Alzheimer’s disease (AD) is a progressive neurodegenerative disorder afflicting an increasing number of elderly people.^1^ The neuropathological hallmarks of AD include the presence of senile plaques (a.k.a amyloid plaques) in the cerebral cortex and hippocampus.^2^ These plaques, which are mainly composed of extracellular aggregates of amyloid-β peptides (Aβs), have been hypothesized to initiate a pathological cascade that eventually results in cognitive decline.^3,4^ There is strong evidence for a causative role of Aβ and its derivatives in Alzheimer’s disease (AD), including human genetics of familial AD (FAD)^5–7^ and Down’s syndrome (DS),^8,9^ toxicity of Aβ aggregates,^10^ Aβ activation of neuron inflammation^11^ and potentiation of tau pathology.^12^ Studies of DS patients are especially convincing. The APP gene resides on chromosome 21. Trisomy 21, a.k.a. DS, almost invariably leads to AD at an early age. Strikingly, three DS patients with partial trisomy that excludes the APP gene did not develop dementia,^13,14^ affirming the central role of Aβ and indicating that reduction of amyloid load is a fundamentally sound disease-modifying strategy. Very recently, aducanumab, a monoclonal antibody against Aβ developed by Biogen, was announced to cause a significant reduction in cognitive decline at the highest dose (10 mg kg^−1^), encouraging further development of anti-Aβ therapies.

γ-Secretase cleaves within the transmembrane domain of the amyloid precursor protein (APPTM) to release Aβ from C99, which aggregates to form neurotoxic oligomers and fibrils (Fig. 1). Thus, the APPTM/γ-secretase interface is an obvious drug target for reducing the amyloid load. We have previously solved the solution NMR structure of APPTM in micelles and have shown that familial AD (FAD) mutants of APPTM, V44M and V44A enhance the flexibility and accessibility of the initial ε-cleavage site for Aβ42 production in APPTM, leading to an increased Aβ42/Aβ40 ratio.^5^ In a subsequent study, we showed that the C-terminal lysine cluster of APPTM participates in the initial docking of APPTM to intramembrane protease, coupled with helical unwinding to prime the substrate for peptide bond hydrolysis.^26^ Recently, in agreement with our NMR studies, the cryo-EM structure of the APP substrate and the γ-secretase complex revealed an α-helical to β-strand transition at the C-terminus of APPTM (shown in Fig. 1).^17^ These studies point to the C-terminal region of APPTM as a promising and novel target to inhibit γ-secretase cleavage of APP.

In *silico* docking, carried out using the solution NMR structure of APPTM as a target, yielded ~60 compounds. Among these, compound C1 was predicted to bind to a pocket at the C-terminus of APPTM and interacts with residues including K53 (Fig. S1 and S2, ESI†). Consistently, C1 caused significant chemical shift perturbations (CSPs), and a large decrease in peak intensity in the 2D ^1^H–^15^N TROSY of APPTM (Fig. 2; for the structure of C1, see Fig. 3). The largest peak intensity changes occurred at residues M51 to K55, indicating that C1 interacts mainly with the C-terminal region of APPTM (Fig. 2a).

To demonstrate that C1 interaction with the substrate can inhibit γ-secretase cleavage of APP, AlphaLISA assays were employed with γ-secretase in the HeLa membrane^18,19^ and biotinylated Sb4 based on the sequence of APP as the substrate.^19^ C1 decreased the production of Aβ40 and Aβ42 by γ-secretase in a dose dependent manner (Fig. 2b). An IC_50_ value of 1.9 μM was obtained for the inhibition of Aβ42 production and 3.9 μM for the inhibition of Aβ40 production. The lower IC_50_ of C1 for Aβ42 production compared to Aβ40 indicated that C1 has selectivity in inhibiting Aβ42 production over Aβ40. Similar inhibition effects were also observed in a gel-based assay (Fig. S3, ESI†) using MBP-APPTM fusion protein as the substrate and the presenilin homolog (PSH) MAMRE50 as an enzyme.^16^ PSHs are archaeal homologs of presenilin and recapitulate important biochemical and structural features of presenilin,^15,20,21^ which is the catalytic subunit of γ-secretase.

The effect of C1 treatment on Aβ40 and Aβ42 production by γ-secretase was then tested in human embryonic kidney 293 (HEK 293) cells using a sandwich ELISA assay. HEK 293 cells were transfected with a plasmid to express human APP695, and Aβ40 and Aβ42 levels were measured in the conditioned medium. After the treatment by 10 μM and 25 μM of C1 for 24 h, the amount of Aβ40 decreased by ~25% (Fig. 2c). In contrast, Aβ42 decreased by ~30% after treatment by 10 μM C1 for 24 h, and by ~70% after treatment by 25 μM C1. In agreement with the AlphaLISA data, C1 reduces the Aβ42 level more than that of Aβ40 in a cellular environment, reducing the Aβ42/Aβ40 ratio.

Using mass spectrometry, we demonstrated that C1 covalently modifies APPTM. MALDI-TOF-MS spectra of APPTM incubated with C1 were recorded (Fig. 3a). Multiple peaks with an interval of ~148 Da were observed after incubation with C1 for 4 h, demonstrating covalent modification of APPTM by C1. The extent of C1 modification increased with C1 concentration and incubation time. At the APPTM:C1 molar ratio of 1 : 1, no modification was observed at 0 h (immediately after mixing) and up to two modifications were found after 4 h of incubation. At an APPTM: C1 ratio of 1 : 5, one modification was observed at 0 h and up to five modifications were detected at 4 h.

Saturation transfer difference (STD) NMR was employed to test whether C1 also interacts with APPTM non-covalently (Fig. 3b). The STD NMR experiment relies on ligand exchange between the bound and free state. In STD NMR, a selective pulse was applied to saturate only the protein resonances. The transfer of this saturation by intermolecular cross relaxation to a bound small molecule is detected by difference spectroscopy.^22^ The STD spectrum of C1 in the presence of APPTM has clear signals (Fig. 3b), while no signal was observed in the absence of APPTM. STD signals can be observed only when a small molecule comes off from the protein-binding site. For covalent binding, once the ligand is covalently attached to the protein, it can no longer exchange or contribute to saturation transfer. Thus, no STD signal can be detected from covalent modification. Therefore, the STD signal we observed indicates that C1 also binds APPTM in a non-covalent manner.

The α,β-unsaturated ketone moiety in C1 is a well-known Michael’s acceptor.^23^ The electron-deficient β-carbon reacts with nucleophiles in proteins, such as the amino group in the side chain of lysine residues.^23^ According to NMR titration (Fig. 2a and 4a), C1 interacts with APPTM at the C terminal region surrounding the juxtamembrane lysines (K53, K54, and K55). Within the APPTM sequence, there are no other strong nucleophiles, except lysine side chains. To test whether C1 can indeed modify the lysine sidechain, free Fmoc-Lysine (Fmoc-Lys) was incubated with C1. C1-Modified Fmoc-Lys (Fmoc-Lys-C1) was separated and detected by LC-ESI-MS with a *Δ*M of 148.0318 Da compared to Fmoc-Lys (Fig. 4b), which is consistent with the *Δ*M of APPTM caused by C1 (Fig. S5a, ESI†). Fmoc-Arg was also tested, but the C1-adduct was not observed for Arg (Fig. S5b, ESI†). When C1 carries out an electrophilic attack at the amino group of the lysine side chain, a naphthalene group should be released as 2-naphthol, which was verified by ^1^H NMR (Fig. 4b).

To further confirm the mechanism of C1 modification, the reactivity of C1 analogs (Fig. 4c) was tested by MALDI-TOF-MS. The fluorine (−F) moiety in C1 was replaced by a methyl group (−CH_3_) and a methoxy group (−OCH_3_) in analog 1 and analog 2, respectively. Analog 1 and 2 showed similar modification patterns as C1 with the expected MW change, while analog 3, in which the Michael’s acceptor is missing, cannot modify APPTM, as expected. The importance of the neutral amine group of the lysine sidechain in this reaction was demonstrated by the pH dependence of C1 modification (Fig. S6a, ESI†). Reduced activity was observed at lower pH, because the lower the pH, the lower the population of the neutral amine group of the lysine sidechain. Based on these results, a reaction mechanism is proposed for the modification of lysine sidechains in APPTM by C1 (Fig. 4d).

Gel-based cleavage assays showed that the C-terminal lysine cluster, in particular K55, plays an important role in the cleavage of APPTM by PSH (Fig. S7, ESI†). To assess the relative roles of different APPTM lysine residues in C1 interaction, we generated five lysine-to-alanine single mutants (K16A, K28A, K53A, K54A, and K55A), and a C-terminal triple-lysine mutant (KKKAAA). In MALDI-TOF-MS, C1 modified K28A and K54A in a similar manner as the WT, while less C1 modification was observed in K55A (Fig. 5a). These data indicated that K55 is the most reactive lysine in C1 modification, likely more accessible and with significantly reduced p*K*_a_ due to proximity to nearby positive charges. Even less C1 modification was observed in the KKKAAA triple mutant, which again demonstrates that C1 mainly interacts and modifies the C-terminal three lysines in APPTM.

A major concern for the covalent modifier is its non-specificity and off-target effects. To access the selectivity of C1 interaction, ubiquitin (with 7 lysines in its sequence and key surface exposed lysines for ubiquitination) and APPTM (5 lysines) were co-incubated with C1 at 40 °C for 4 h. As shown in Fig. 5b, multiple modifications of APPTM were observed while no significant modification was observed for ubiquitin, indicating that C1 selectively modified APPTM in the presence of ubiquitin. The preference of C1 for APPTM over ubiquitin is likely due to the non-covalent binding between C1 and APPTM, and the higher reactivity of juxtamembrane lysines in APPTM *(e.g.* K55).

## Communication

Several compounds have been reported to bind to APP and inhibit Aβ production, but none has been reported to target the C-terminal juxtamembrane region of APPTM. Peptides binding to the N-terminus of C99 have been shown to inhibit Aβ production in a substrate-specific manner.^24^ γ-secretase modulators (GSMs, such as fenofibrate and tarenflurbil) were initially reported to bind to APPTM,^25^ while no specific binding between GSM and APPTM was found in further studies^26^ or in our own hands (data not shown). The anti-cancer drug bexarotene can reduce amyloid load and alleviate neurodegeneration,^27^ but a recent study showed that bexarotene inhibits γ-secretase with low efficacy and this effect is not due to substrate binding.^28^

Here, using NMR, MS, AlphaLISA and cell assays, we discovered a novel compound C1 which binds C-terminal juxtamembrane lysines of APPTM and inhibits γ-secretase production of Aβ. The C-terminal juxtamembrane lysine cluster (K53, K54 and K55) is near the ε-cleavage sites T48 and L49, where the initial cleavage by presenilin occurs.^29^ The inhibition of C1 on γ-secretase cleavage may be rationalized by the recent cryo-EM structure of the complex of the APP C83 substrate and γ-secretase. In this structure, the C-terminal α-helix in APPTM unwinds into an extended β-conformation to expose the ε-cleavage sites,^17^ forming an intermolecular β-sheet with two β-strands from PS1. The C1 modification of the C-terminal juxtamembrane lysines of APPTM likely interferes with the α to β conformational transition and/or the formation of the intermolecular β-sheet between APPTM and PS1, inhibiting γ-secretase cleavage.

Our study provides the first proof-of-concept that targeting the C-terminal juxtamembrane lysines of APPTM is sufficient for reducing Aβ production, pointing to a new direction in AD drug discovery for reducing the amyloid load as disease-modifying therapy.

## Supplementary Material

SI

## Figures and Tables

**Fig. 1:**
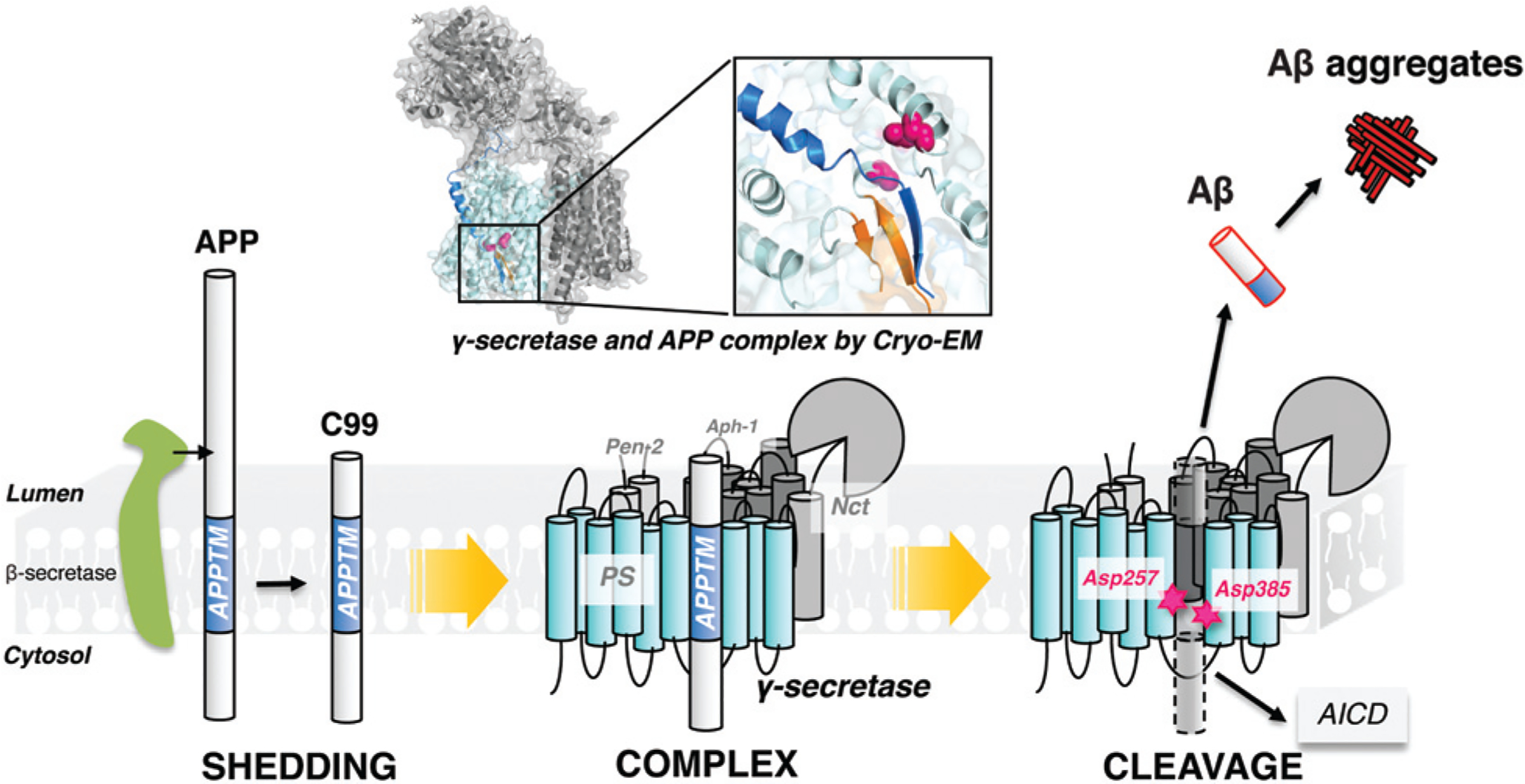
γ-Secretase cleaves APP within the transmembrane domain to generate the C-terminus of Aβ, releasing it from the plasma membrane.

**Fig. 2:**
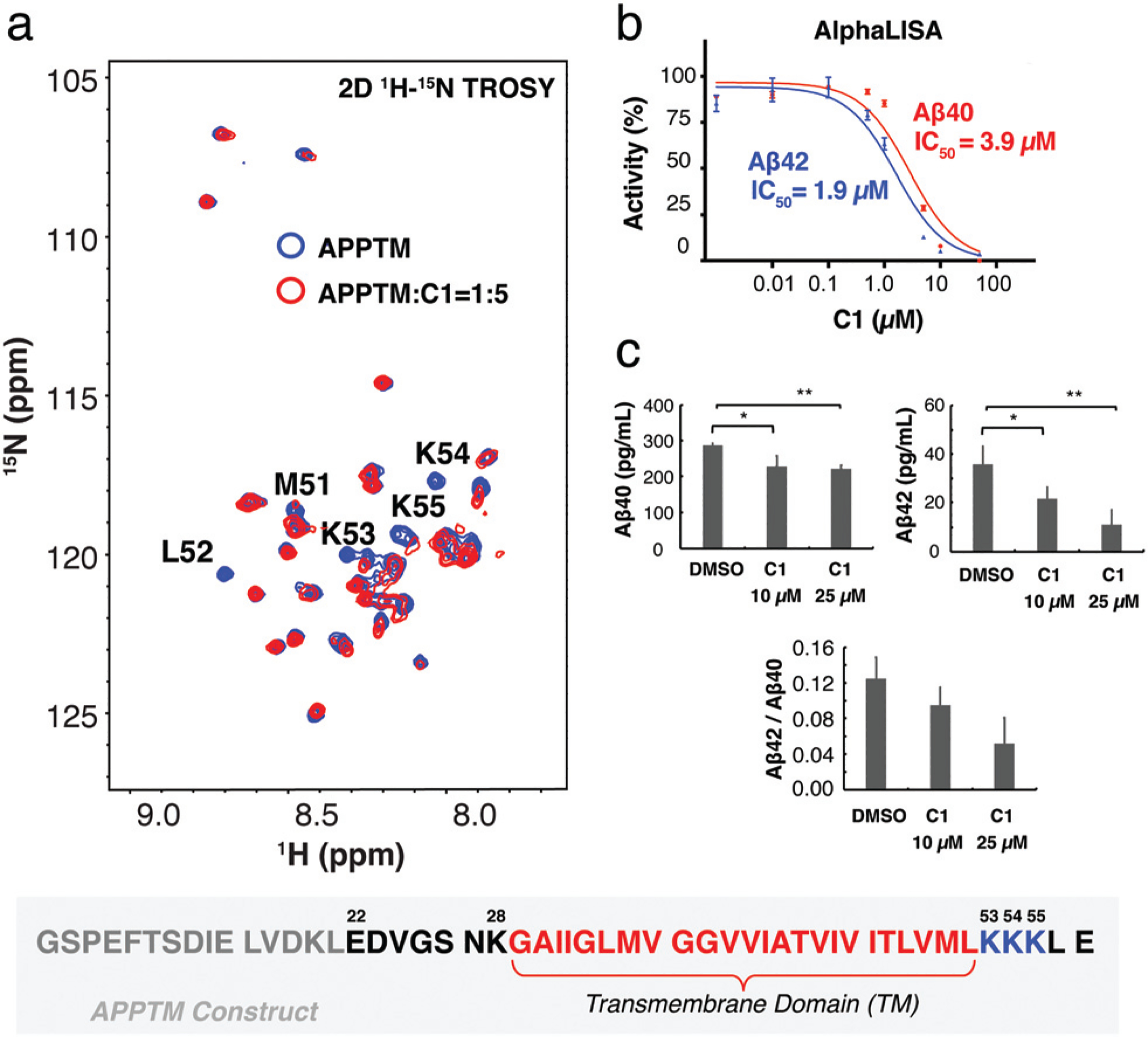
C1 interacts with APPTM C-terminal lysines and inhibits γ-secretase cleavage in both biochemical and cellular assays. (a) Overlay of the 2D ^1^H–^15^N TROSY spectrum of APPTM with (red) and without C1 (blue). Resonances with the largest changes in peak intensity were labeled by residue type and number. The sequence of the APPTM construct is shown below the spectra, with APP transmembrane domain residues in red, juxtamembrane lysines in blue, additional APP residues in black, and non-APP residues in grey. (b) C1 reduces the production of Aβ40 and Aβ42 by γ-secretase with an IC_50_ of 3.9 μM and 1.9 μM, respectively, in an AlphaLISA γ-secretase assay. (c) C1 reduced the level of Aβ40, Aβ42 and Aβ42/Aβ40 ratio in HEK 293 cells. Aβ40, *(*p* = 0.0260, unpaired *t*-test, *n* = 3), **(*p* = 0.0007, unpaired *t*-test, *n* = 3). Aβ42, *(*p* = 0.047, unpaired *t*-test, *n* = 3), **(*p* = 0.028, unpaired *t*-test, *n* = 3).

**Fig. 3:**
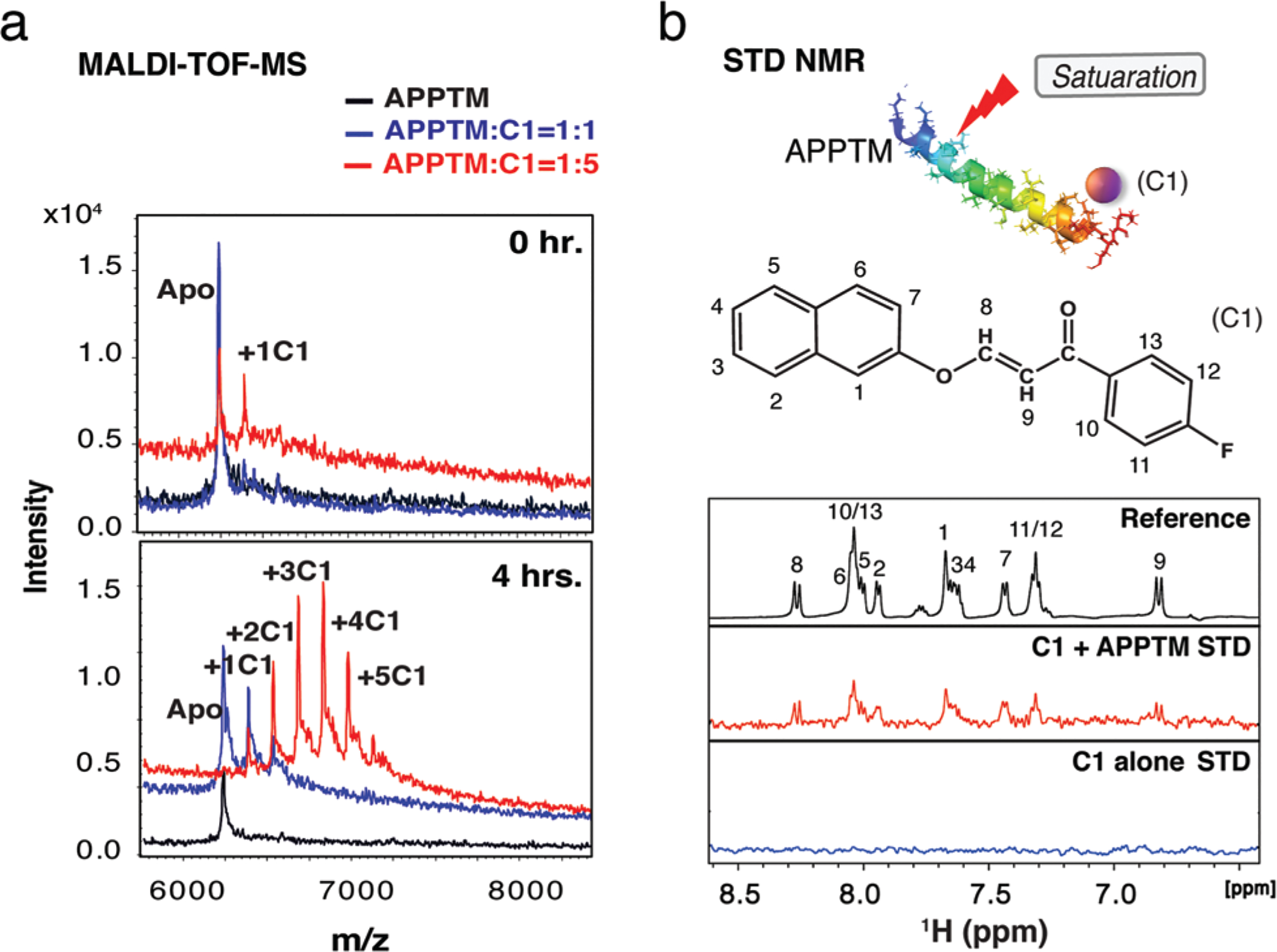
C1 interacts with APPTM both covalently and noncovalently. (a) MALDI-TOF-MS showed that C1 covalently modifies APPTM in a dosage- and time-dependent manner. (b) STD NMR demonstrated that C1 also binds non-covalently to APPTM. The assignment of C1 shown in the reference spectrum (APPTM not saturated by NMR pulses) was achieved by analyzing a series of 2D experiments including ^1^H–^1^H COSY, ^1^H–^1^H TOCSY, ^1^H–^13^C HMQC and ^1^H–^13^C HMBC (Fig. S4, ESI†).

**Fig. 4:**
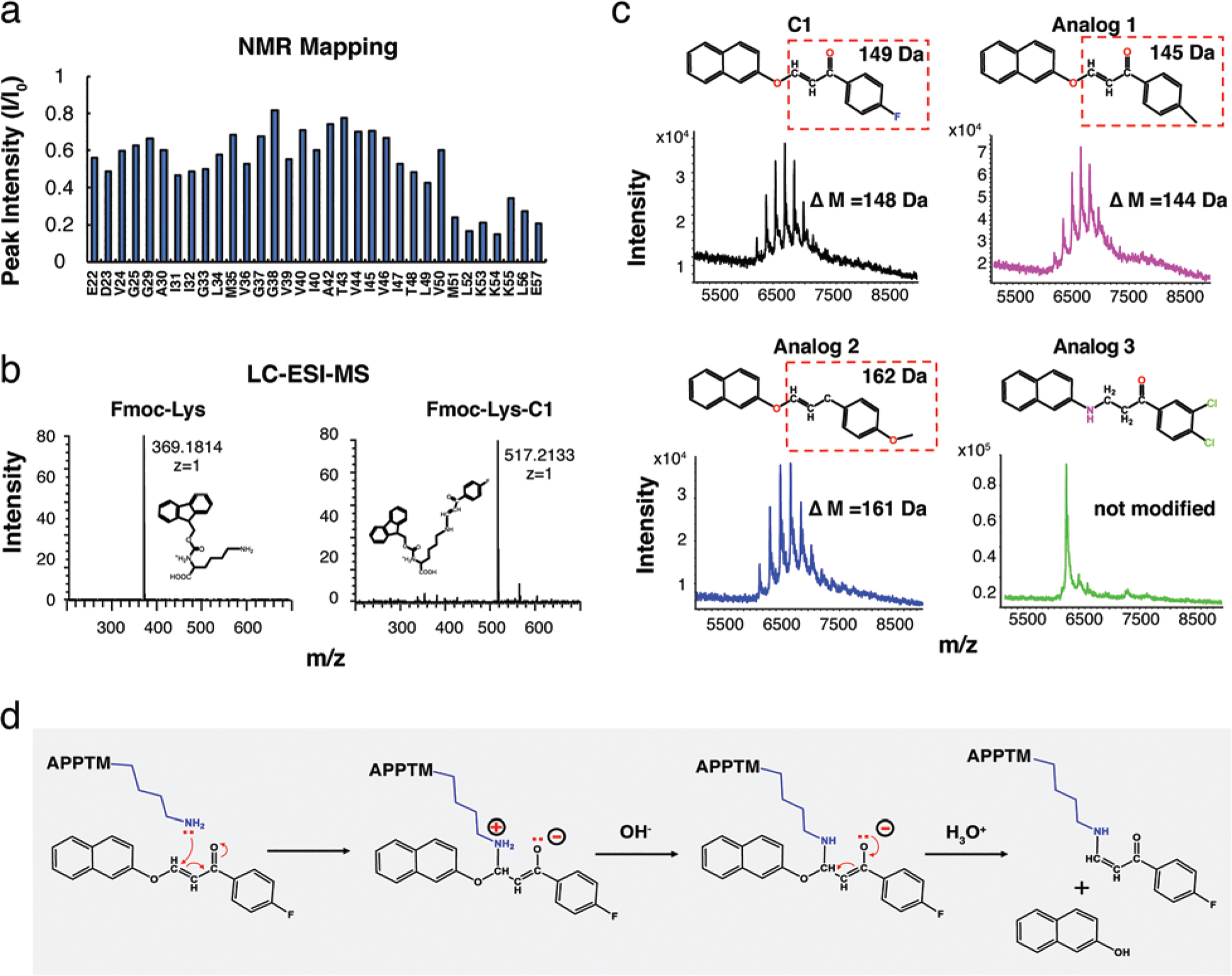
C1 covalently modifies C-terminal juxtamembrane lysine side chains of APPTM by electrophilic attack. (a) C-terminal residues from M51 to E57 exhibited the largest peak intensity decrease in NMR titration. (b) C1 modifies free Fmoc-Lys with the same Δ*M* as it modifies APPTM. (c) Activity of C1 analogs towards APPTM detected by MALDI-TOF-MS, with an expected molecular weight change (Δ*M*). (d) Mechanism for the modification of the lysine side chain in APPTM by C1 through electrophilic attack.

**Fig. 5:**
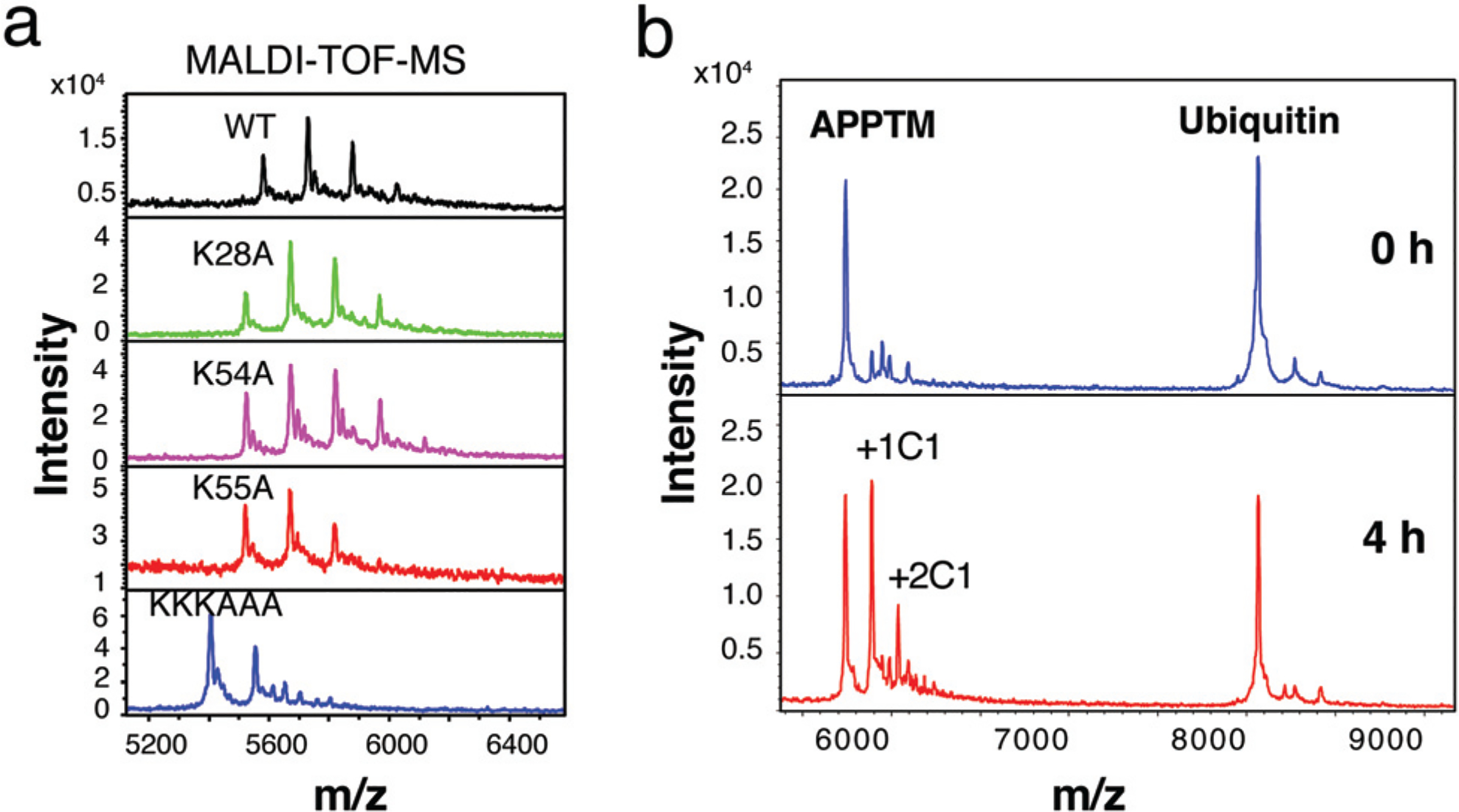
K55 plays an important role in C1 modification and C1 selectivity of APPTM over ubiquitin. (a) C1 modifies K28A and K54A similarly as WT, while less modification was observed in K55A. (b) C1 selectively modified APPTM (5 lysines) in the presence of ubiquitin (7 lysines).
