# Supplementary material for: Substrate interaction inhibits γ-secretase production of amyloid-β peptides
Source: Chem Commun (Camb). Author manuscript; Available in PMC 2021 Jun 22. (PMC8219260; doi:10.1039/c9cc09170j)
Supplement: SI [file NIHMS1676708-supplement-SI.pdf]

Electronic Supplementary Material (ESI) for ChemComm.
This journal is © The Royal Society of Chemistry 2020

Substrate Interaction Inhibits γ-secretase Production of Amyloid-β Peptides

(Supporting Information)

*Jing Zhao, Yuanyuan Xiao, Xinyue Liu, Soohyun Kim, Xianzhong Wu, Marilia Barros, Ran Zhuang, Xuben Hou, Yingkai Zhang, Nikolaos K. Robakis, Yue-Ming Li, Jonathan S. Dordick, Iban Ubarretxena-Belandia, Chunyu Wang**

Figures

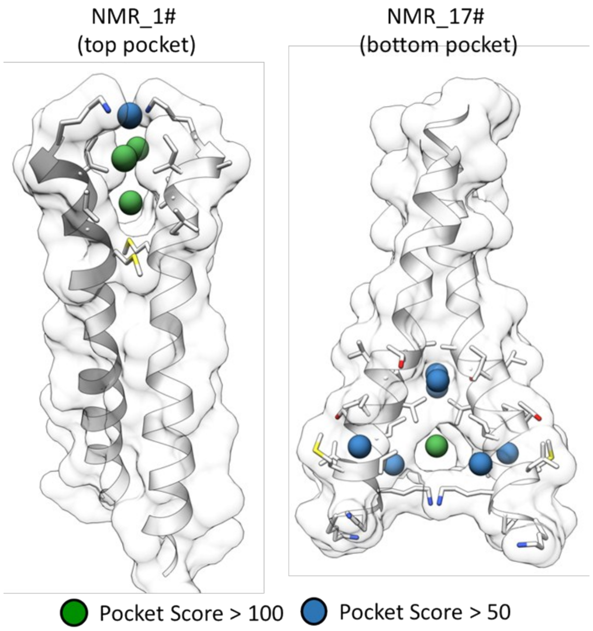

Figure S1. Binding pockets in APPTM calculated by AlphaSpace. AlphaSpace was used to calculate the potential binding pockets in APPTM based on our previously reported NMR structure (PDB: 2LZ3). We found two major binding pockets at the top and bottom of the APPTM helical dimer. Next, AutoDock Vina was employed for virtual screenings of the SPECS commercial database (www.specs.net). The top-scoring compounds were selected to for evaluation by NMR.

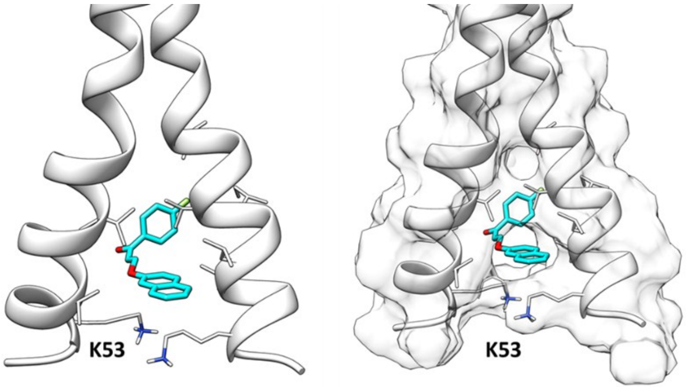

Figure S2. Docking pose of C1 in APPTM. Compound C1 binds to the bottom pocket of APPTM and interacts with residues including K53.

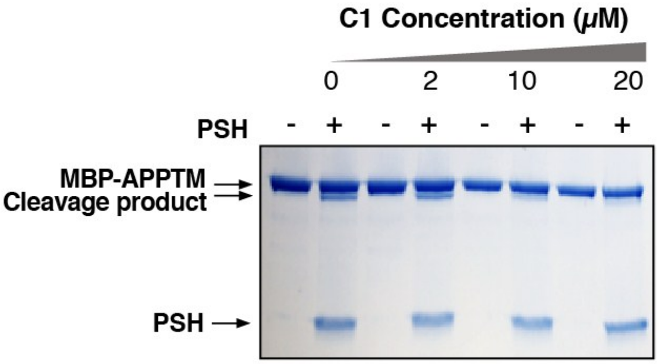

Figure S3. C1-modified substrate inhibits the cleavage of APPTM by PSH MAMRE50 in a gel-based assay. An MBP-APPTM fusion protein was used as the substrate and a presenilin homolog (PSH) MAMRE50 [1] was used as the enzyme. After the substrate incubation with PSH, a band with smaller molecular weight was observed, demonstrating the intramembrane proteolysis of APPTM [1,2]. Weaker cleavage bands were observed for C1-modified MBP-APPTM, indicating significant inhibition on PSH cleavage. At 20 μM C1, cleavage was almost completely blocked. To avoid the complication that C1 may interact with the enzyme to inhibit its activity, C1 was incubated with the substrate and then removed before cleavage assay.

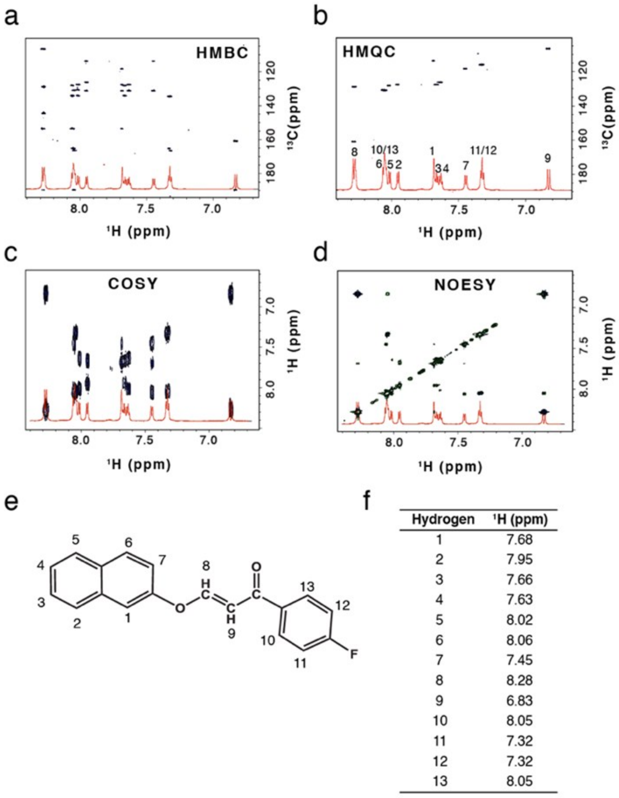

Hydrogen	^{1}H (ppm)
1	7.68
2	7.95
3	7.66
4	7.63
5	8.02
6	8.06
7	7.45
8	8.28
9	6.83
10	8.05
11	7.32
12	7.32
13	8.05

Figure S4. **Assignment of protons in compound C1** by 2D ^{1}H-^{13}C HMBC (a), ^{1}H-^{13}C HMQC (b), ^{1}H-^{1}H COSY (c), and ^{1}H-^{1}H NOESY (d). The numbering of protons in C1 and chemical shift of each proton were shown in e and f, respectively.

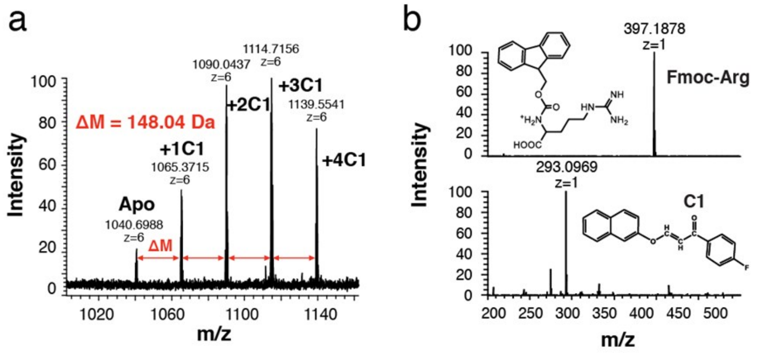

Figure S5. **Evidences for mechanism of C1 modification by LC-ESI-MS.** a) Precise molecular weight difference between APPTM-C1 adducts (ΔM) was detected to be 148.04 Da. b) C1 doesn't modify free Fmoc-Arginine (Fmoc-Arg).

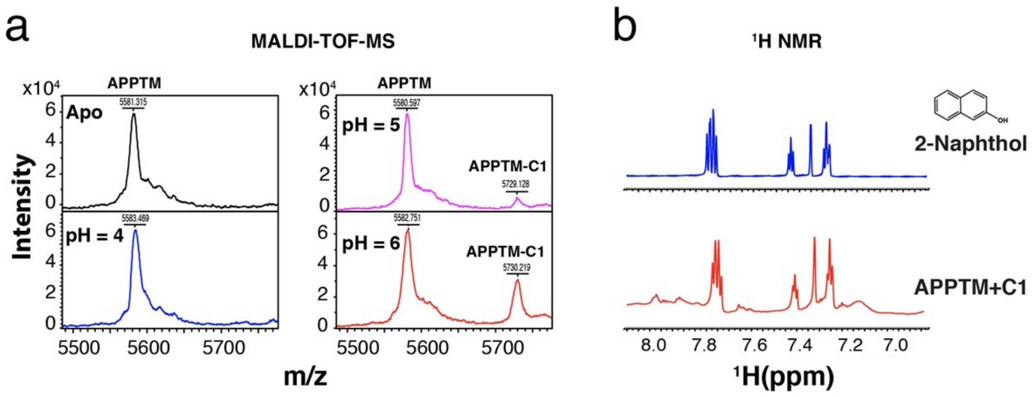

Figure S6. pH dependence and release of 2-naphthol in C1 modification. a) C1 modifies APPTM at pH 6.0, while modification at pH 5.0 was significantly decreased, and no modification was observed at pH 4.0. b) Release of 2-naphthol from APPTM-C1 reaction verified by 1D ^{1}H NMR spectra of 2-naphthol and APPTM-C1 mixture.

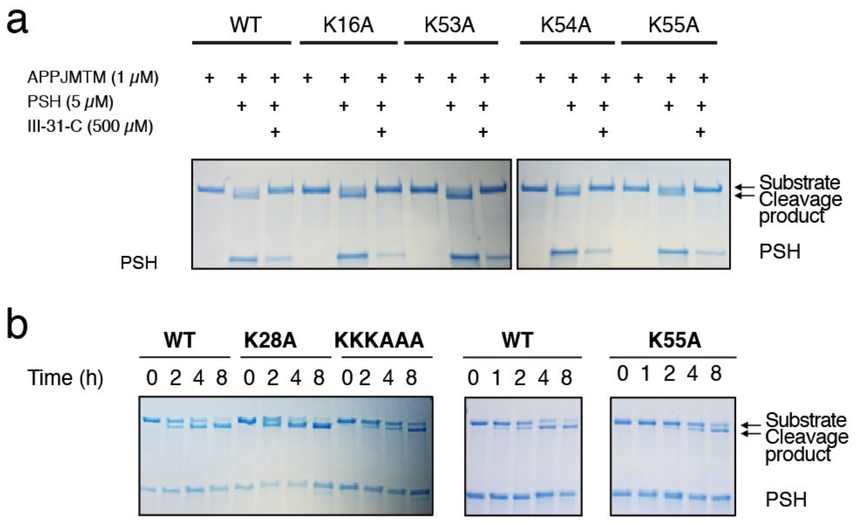

Figure S7. **K55 plays an important role in intramembrane proteolysis of APPTM.** a) Cleavage of lysine mutant K55A by PSH MAMRE50 was significantly reduced compared to WT and other mutants. b) Mutant K55A and KKKAAA exhibit significantly reduced cleavage by PSH MAMRE50 in a time-course assay.

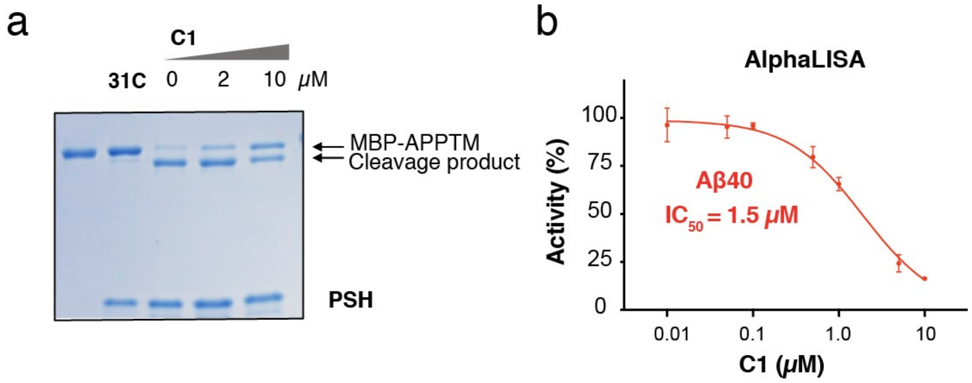

Figure S8. C1 inhibits intramembrane proteolysis of APP with μM $IC_{50.}$ (no pre-incubation). a) Inhibition of C1 on intramembrane proteolysis of APP by a gel-based assay using purified PSH MAMRE50. A known γ-secretase inhibitor III-31C [3] was used as a positive control, showing clear inhibition (no band for cleavage product) on the APPTM cleavage by PSH. Significant inhibition on cleavage was observed in the presence of C1, shown by a significantly weak band for cleavage product at 10 μM. b) An IC_{50} of 1.5 μM was obtained for the inhibition of Aβ40 production in AlphaLISA assay using APP as substrate and γ-secretase in Hela membrane**.**

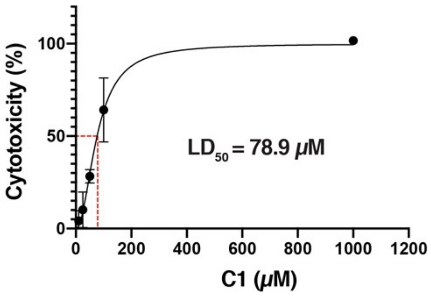

Figure S9. Cytotoxicity of C1 on HEK293 cells transduced with APP695. Lethal dose (LD_{50}) was measured to be 78.9 μM when HEK 293 cells transduced with APP695 were treated with C1 for 24 h. Cytotoxicity of C1 was tested at the concentrations of 10, 25, 50, 100 and 1000 μM. The mean of three measurements was used to draw % of toxicity graph.

Materials and Methods

Protein expression and purification.

The overexpression and purification of APPTM were described previously by our group [4] and others [5,6]. Using the pETM41 vector, the MBP-APPTM-His_6 fusion protein was successfully overexpressed in *E. coli* BL21 DE3 Condon Plus RIPL cells. The fusion protein was used as the substrate in gel-based cleavage assay by PSH MAMRE50. The fusion protein was cleaved by thrombin to generate isotopically labeled APPTM for NMR studies. APPTM was reconstituted into micelles to yield high quality 2D ^{1}H-^{15}N TROSY spectra. Lysine mutants of APPTM were overexpressed and purified using the same protocol with modified plasmids created by site directed mutagenesis. The presenilin homolog (PSH) MAMRE50 was overexpressed and purified using the protocol by J.W. Cooley et al.[7] .

Solution NMR

Following purification, APPTM was buffer exchanged into NMR buffer (25 mM sodium phosphate, pH=7.2) where the final dodecyl phosphocholine (DPC) (Anatrace, Maumee, OH) concentration was adjusted to 4%. Well-resolved ^{1}H-^{15}N TROSY spectra were recorded in 10% D_2O on an 800 MHz Bruker Advance II spectrometer equipped with cryogenic probes. The assignment of the ^{1}H-^{15}N TROSY spectrum of APPTM was previously accomplished and deposited in BMRB (Entry 18649) [8].

Compound C1 was added to ^{15}N-labeled APPTM sample at a ratio of 1:5. ^{1}H-^{15}N TROSY spectra were collected for APPTM before and after adding C1 at 323K. The spectra were analyzed using Sparky (T.D. Goddard AND D.G. Kneller, SPARKY 3, University of California, San Francisco, CA) and Microsoft Excel. The intensity changes of each resonance peak caused by C1 were calculated by I/I_0, where I and I_0 represent the peak intensity after and before C1 addition, respectively. The calculated I/I_0 was plotted against amino acid residues from the N- to C- terminus

of APPTM to map the binding site. 1D ^{1}H spectrum of 2-naphtol was recorded in same NMR buffer and compared with the spectrum of APPTM-C1 mixture.

MALDI-TOF-MS and LC-ESI-FTMS

MALDI-TOF-MS and LC-ESI-FTMS were applied for the detection of C1 modification. All reactions were carried out using ^{15}N labeled APPTM in NMR buffer. The DPC in samples was removed by a detergent removal spin column (Pierce, · Rockford, IL,) before MALDI or ESI detection. MALDI-TOF-MS spectra of APPTM were acquired on a Bruker Daltonics-autoflex™ speed MALDI-TOF/TOF spectrometer. A sensitive linear mode was applied for detecting APPTM with sinapinic acid as the matrix. A Thermo Scientific LTQ ORBITRAP XL spectrometer coupled with an Agilent 1200 HPLC was used to acquire the high-resolution mass spectra. Data were processed in Thermo Xcalibur 2.2.

C1 modification by MS. MALDI-TOF-MS spectra of APPTM (6241 Da, 50 μM) were recorded with 1:1 and 1:5 ratio of C1 treatment at timepoints of 0 h and 4 h. Exact mass change of APPTM by C1 modification was detected and calculated by LC-ESI-FTMS. A Waters MassPrep, Phenyl Guard Column (2.1 mm X 10 mm) was used to desalt and concentrate the injected samples. Mobile phase A is water with 0.1% FA, mobile phase B is 95% acetonitrile with 0.1% FA. Flow rate is 0.3 mL/min. A fast gradient of 5% to 90% B from 3 to 8 min was applied. To get a steady ion current, the ion source was tuned (Spray voltage 4.8 kV, sheath gas flow 40 arb, aux gas flow 20 arb, capillary temperature 275 ℃, tube lens 175 V). The mass spectra were collected under positive mode of 60,000-resolution using FTMS analyzer, and the mass range was defined as 200-2000 m/z.

C1 modification of Fmoc-lysine and Fmoc-arginine by MS. 50 μM of Fmoc-lysine and Fmoc-arginine were dissolved in the NMR buffer, separately, and incubated with a 1:2 ratio of C1 at 40 °C for 4 h before detection by LC-ESI-FTMS. An Agilent Poroshell 120 SB-C18 (2.1 x 150 mm, 2.7 μm) column was applied for LC separation. Mobile phase A is water with 0.1% FA, mobile

phase B is 95% acetonitrile with 0.1% FA. Flow rate is 0.3 mL/min. A gradient of 5% to 90% B from 5 to 18 min was applied. Mass spectra were collected under positive mode. The extracted m/z values are: 293.09-293.10 for C1 molecule, 369.17-369.19 for Fmoc-lysine, 517.20-517.23 for C1 modified Fmoc-lysine, 397.17-397.19 for Fmoc-arginine, 545.21-545.23 for C1 modified Fmoc-arginine. Fmoc-lysine and Fmoc-arginine solution without C1 were detected as negative controls.

C1 analogs activity. The modification activity of three analogs of C1 was tested on APPTM by MALDI-TOF-MS and compared to C1. 50 μM of APPTM was mixed with 1:5 ratio of C1 and different analogs and incubated at 40 °C for 4 h before detection.

pH dependence. The capacity of C1 modifies APPTM (5581 Da, 50 μM) at different environmental pH was tested using MALDI-TOF-MS. The pH of APPTM solution was adjusted to 4.0, 5.0, and 6.0, separately, by an Orion Star™ A111 pH Benchtop Meter equipped with a pH micro electrode (Fisher Scientific, Fair Lawn, NJ). 50 μM of APPTM at different environmental pH was incubated with 1:2 ratio of C1 at 40 °C for 4 h and detected by MALDI-TOF-MS.

C1 selectivity. 50 μM (final concentration) of APPTM and 50 μM of ubiquitin were mixed together before incubation with a 1:4 ratio of C1 at 40 °C. Spectra of the mixture at two timepoints after C1 addition, 0 h and 4 h, were recorded and analyzed.

STD NMR

STD spectra were recorded at 313K on a Bruker 600 MHz NMR spectrometer. 1 mM C1 was mixed with 10 μM APPTM, dissolved in NMR buffer. 1D STD-NMR spectra were recorded with 1440 scans and selective saturation of protein resonances at 0 ppm using a series of Gaussian shaped pulses, for a total saturation time of 2.0 s. For certain experiments, a 30 ms spin lock was also employed to suppress peptide signals that overlap C1 resonances. Saturation transfer reference (STR) spectrum was recorded by off-resonance frequency set to -10.0 ppm. STD spectrum of 1mM C1 in the absence of APPTM was conducted under the same parameters as a negative control.

Gel-based cleavage assay by PSH MAMRE50

A gel-based assay was established to test the cleavage of APPTM and C1-modified APPTM by PSH MAMRE50. The fusion protein MBP-APPTM (1 μM) was used as the substrate and modified by different ratio of C1 (1:2, 1:10, and 1:20) before cleaved by PSH. Residual C1 and reaction product 2-naphthol were removed by a desalting column. The native and C1-modified substrate were incubated with 5 μM of PSH at 37° for 24 h before running on a 12% SDS-PAGE gel. III-31-C, a known γ-secretase inhibitor, was pre-incubated with PSH for 1h before adding the substrate.

For the substrate competition assay, different amounts (0.5, 1, 2, 5 or 10 μM) of C1 modified MBP-APPTM were added to 1 μM native substrate and 5 μM PSH mixture, and incubated at 37 °C for 24 h before running on a 12% SDS-PAGE gel. Same protocol was used for the gel-based cleavage assay of lysine mutants.

γ-secretase cleavage by AlphaLISA assay

A well-established AlphaLISA assay [9–11] was employed to test if C1-modified substrate can inhibit the γ-secretase cleavage *in vitro*. HeLa-S3 cell was purchased from National Cell Culture Center and solubilized membrane was prepared as previously described [10,12]. The Sb4 substrate based on the sequence of APP (1 μM) was incubated with 1:0.001, 1:0.01, 1:0.1, 1:0.5, 1:1, 1:5, 1:10 and 1:50 ratio of C1 at 37 °C for 4 h. After shaking with solubilized γ-secretase (40 μg/mL) at 110 rpm for 3 h at 37 °C, the AlphaLISA detection mixture that includes Aβ40 and Aβ42 antibody (G2-10 and 10G3, respectively), protein A-conjugated acceptor beads, streptavidin-conjugated donor beads were added and incubated overnight. The AlphaLISA signal was detected using Envision plate reader (Perkin Elmer, Waltham, MA). Data were processed using GraphPad Prism (version 8.1.0). IC_{50} value was fitted by a non-linear regression equation:

$$Y = Y_0 + (Y_m - Y_0) / (1 + 10\wedge(X - Log^{IC50}))$$

where Y_m and Y_0 represent the highest and lowest Aβ level, respectively, and X is C1 concentration.

Cell culture methods

HEK293 cells were stably transfected with human APP695 in pMX-IRES-GFP vector purchased from GenScript, Piscataway, NJ as described previously [13]. HEK293 expressing APP695 were grown in Dulbecco's modified Eagle's medium plus 10% fetal bovine serum, penicillin and streptomycin. For both toxicity assay and soluble Aβ level detection, the medium was collected after APP695-HEK293 cells were treated with C1 for 24 h Cell viability was evaluated by the 3-[4,5-dimethy-2-thiazolyl)-2,5-diphenyl-2H-tetrazolium bromide (MTT) reduction method using cell proliferation kit (Sigma-Aldrich, St. Louis, MO). The absorbance at 570 nm (formazan product) minus the absorbance at 630 nm (background) was recorded according to manufacturer's instruction. The corrected absorbance was used to calculate the percent cell viability by using the following formula for each sample: (corrected Absorbance of experiment-corrected Absorbance of blank)/(corrected Absorbance of DMSO control-corrected Absorbance of blank)*100. The concentration of Aβ in conditioned medium was measured by Aβ40 and Aβ42 specific ELISAs (Life Technologies, Carlsbad, CA), respectively, as described previously [14]. The absorbance was determined for each well at 450 nm using a microplate reader (Bio-Rad, Hercules, CA).

References

1 N. Clemente, A. Abdine, I. Ubarretxena-Belandia and C. Wang, *Sci. Rep.*, 2018, **8**, 4–11.

2 C. Torres-Arancivia, C. M. Ross, J. Chavez, Z. Assur, G. Dolios, F. Mancia and I. Ubarretxena-Belandia, *PLoS One*, 2010, **5**, 1–11.

3 W. P. Esler, W. T. Kimberly, B. L. Ostaszewski, W. Ye, T. S. Diehl, D. J. Selkoe and M. S. Wolfe, *Proc. Natl. Acad. Sci.*, 2002, **99**, 2720–2725.

4 W. Chen, E. Gamache, D. Richardson, Z. Du and C. Wang, *Protein Expr. Purif.*, 2012, **81**, 11–17.

5 K. D. Nadezhdin, O. V Bocharova, E. V Bocharov and A. S. Arseniev, *Acta Naturae*, 2011, **3**, 69–76.

6 O. V. Bocharova, A. S. Urban, K. D. Nadezhdin, E. V. Bocharov and A. S. Arseniev, *Protein Expr. Purif.*, 2016, **123**, 105–111.

7 J. W. Cooley, A. Abdine, M. Brown, J. Chavez, B. Lada, R. D. JiJi and I. Ubarretxena-Belandia, *A New Method to Determine the Transmembrane Conformation of Substrates in Intramembrane Proteolysis by Deep-UV Resonance Raman Spectroscopy*, Elsevier Inc., 1st edn., 2017, vol. 584.

8 W. Chen, E. Gamache, D. J. Rosenman, J. Xie, M. M. Lopez, Y.-M. Li and C. Wang, *Nat. Commun.*, 2014, **5**, 3037.

9 D.-M. Chau, D. Shum, C. Radu, B. Bhinder, D. Gin, M. L. Gilchrist, H. Djaballah and Y.-M. Li, *Comb. Chem. High Throughput Screen.*, 2013, **16**, 415–424.

10 C. C. Shelton, Y. Tian, D. Shum, C. Radu, H. Djaballah and Y.-M. Li, *Assay Drug Dev. Technol.*, 2009, **7**, 461–470.

11 D. M. Chau, C. J. Crump, J. C. Villa, D. A. Scheinberg and Y. M. Li, *J. Biol. Chem.*, 2012, **287**, 17288–17296.

12 Y. M. Li, M. T. Lai, M. Xu, Q. Huang, J. DiMuzio-Mower, M. K. Sardana, X. P. Shi, K. C. Yin,

J. A. Shafer and S. J. Gardell, *Proc. Natl. Acad. Sci. U. S. A.*, 2000, **97**, 6138–43.

13 D. Ito, Y.-M. Iizuka, M. P. Katepalli and K. Iizuka, *Proc. Natl. Acad. Sci.*, 2009, **106**, 11264–11269.

14 S. Dixit, A. Bernardo, J. M. Walker, J. A. Kennard, G. Y. Kim, E. S. Kessler and F. E. Harrison, *ACS Chem. Neurosci.*, 2015, **6**, 570–581.
